# iTRAQ-Based Quantitative Proteomics Analysis Reveals the Mechanism Underlying the Weakening of Carbon Metabolism in Chlorotic Tea Leaves

**DOI:** 10.3390/ijms19123943

**Published:** 2018-12-07

**Authors:** Fang Dong, Yuanzhi Shi, Meiya Liu, Kai Fan, Qunfeng Zhang, Jianyun Ruan

**Affiliations:** 1 Tea Research Institute, Chinese Academy of Agricultural Sciences, Hangzhou 310008, China; 18305811752@163.com (F.D.); shiyz@tricaas.com (Y.S.); liumeiya@tricaas.com (M.L.); fankaitea@tricaas.com (K.F.); jruan@tricaas.com (J.R.); 2 Key Laboratory for Plant Biology and Resource Application of Tea, the Ministry of Agriculture, Hangzhou 310008, China

**Keywords:** *Camellia sinensis*, chlorotic mutation, chlorophyll deficiency, weakening of carbon metabolism, iTRAQ, proteomics

## Abstract

To uncover mechanism of highly weakened carbon metabolism in chlorotic tea (*Camellia sinensis*) plants, iTRAQ (isobaric tags for relative and absolute quantification)-based proteomic analyses were employed to study the differences in protein expression profiles in chlorophyll-deficient and normal green leaves in the tea plant cultivar “Huangjinya”. A total of 2110 proteins were identified in “Huangjinya”, and 173 proteins showed differential accumulations between the chlorotic and normal green leaves. Of these, 19 proteins were correlated with RNA expression levels, based on integrated analyses of the transcriptome and proteome. Moreover, the results of our analysis of differentially expressed proteins suggested that primary carbon metabolism (i.e., carbohydrate synthesis and transport) was inhibited in chlorotic tea leaves. The differentially expressed genes and proteins combined with photosynthetic phenotypic data indicated that 4-coumarate-CoA ligase (4CL) showed a major effect on repressing flavonoid metabolism, and abnormal developmental chloroplast inhibited the accumulation of chlorophyll and flavonoids because few carbon skeletons were provided as a result of a weakened primary carbon metabolism. Additionally, a positive feedback mechanism was verified at the protein level (Mg chelatase and chlorophyll b reductase) in the chlorophyll biosynthetic pathway, which might effectively promote the accumulation of chlorophyll b in response to the demand for this pigment in the cells of chlorotic tea leaves in weakened carbon metabolism.

## 1. Introduction

Tea (*Camellia sinensis*) is a perennial evergreen leafy woody plant native to southwest China. Recently, chlorophyll-deficient chlorina tea plant cultivars have become valuable materials in processing high quality green tea because of their high amino acid content and low catechin content [1,2]. The natural mutant of tea, “Huangjinya”, exhibits chlorotic leaves and lower carbon metabolism than non-chlorotic varieties under sunlight [1,2]. In our previous studies [2,3], metabolomics and transcriptomics analyses were performed on green and chlorotic shoots of “Huangjinya” to gain an overview of the amino acid, flavonoid, and carbohydrate metabolism. These analyses revealed that the weakening of carbon metabolism is accompanied by nitrogen accumulation, suggesting that the metabolism of carbon and nitrogen are unbalanced [3]. Satou et al. [4] have shown similar results in the pale green mutants of *Arabidopsis thaliana*. However, the correlation of protein expression with weakened carbon metabolism in chlorotic tea leaves remains to be elucidated.

Chlorophyll consists of chlorophyll a and chlorophyll b, and plays indispensable roles in harvesting and transferring light energy during photosynthesis and carbon assimilation [5]. Chlorosis in tea leaves has always been attributed to chlorophyll deficiency. Studies on chlorophyll biosynthesis have been widely reported, and at least in angiosperm plants represented by *Arabidopsis thaliana*, genes for all 15 steps in the chlorophyll biosynthesis pathway, starting from the biosynthesis of glutamyl-tRNA to that of chlorophylls a and b, have been identified [6]. However, the effect of chlorophyll metabolism on photosynthesis is largely unclear. Mutants defective in chlorophyll biosynthesis have been identified in higher plants [7,8]. For example, the leaf phenotype was yellow-green in the chlorophyll mutant (*Oryza sativa*) and the level of chlorophyll decreased, meanwhile, chloroplast development was delayed [8]. Thylakoid proteome analysis of a novel rice (*Oryza sativa*) mutant, Zhenhui 249Y, and the wild type has shown that the reduction of chlorophyll b affects the assembly of light harvesting complex I (LHC-I) more severely than that of LHC-II [9].

Proteomics of leaf color mutants of tea plant has been performed using both isobaric tags for relative and absolute quantification (iTRAQ) [10] and two-dimensional gel electrophoresis (2-DE)–mass spectrometry [11,12]. In these studies, 437 differentially accumulated proteins have been identified between the tea plant cultivars “Longjing43” and “Zhonghuang1” [10] and 46 differentially abundant proteins between tender purple and mature green leaves of tea plant [12]. However, it is difficult to clarify the mechanism of weakened carbon metabolism because of the complex genetic background or inconsistent developmental stages of experimental material in different tea varieties [10]. In this study, we used chlorotic and normal green leaves (“Huangjinya”, the albino tea plant cultivar) with the same genetic background and developmental stage as the experimental material to compare the protein expression profiles of shaded and non-shaded leaves by iTRAQ technique.

It is hypothesized that the inhibition of carbon assimilation results in a down-regulation of protein expression in carbohydrate synthesis and transport pathways, further weakening primary carbon metabolism. Meanwhile, flavonoid metabolism, as the major secondary metabolism, may also be suppressed by the down-regulation of the expression of related proteins. In this study, iTRAQ-based quantitative proteomics with phenotypic, biochemical, and transcriptome data confirmed our findings on the differences in protein expression profiles underlying the weakening of carbon metabolism in chlorotic “Huangjinya” tea leaves. The results of this study also provide new insights into the expression level of proteins to understand the mechanisms responsible for chlorophyll deficiency in etiolated tea plant leaves.

## 2. Results

### 2.1. Phenotype, Ratio of Pigment Content, Photosynthesis of Chlorotic and Green Leaves

**Figure 1:**
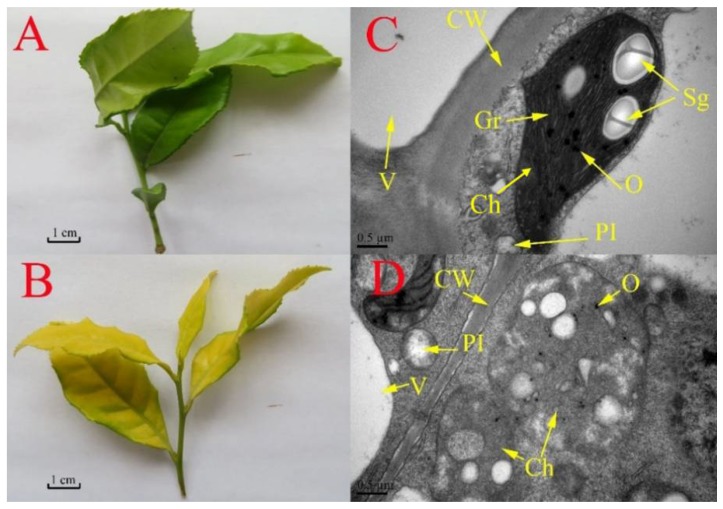
Characterization of the phenotype and ultra-structure of chlorotic and green leaves of the tea plant mutant cultivar “Huangjinya”. (**A**,**B**) Young shoots either grown in shade with 60% light intensity (**A**) or exposed to 100% sunlight (**B**). (**C**,**D**) Ultrastructure of leaves grown under shade (**C**) or under full sunlight (**D**). Ch: chloroplast; CW: cell wall; Gr: grana; O: osmiophilic granules; Pl: plastid; Sg: starch granule; V: vacuole.

Compared with shaded leaves of tea plants (Figure 1A), leaves of tea plants grown under full sunlight were chlorotic and exhibited a yellow phenotype (Figure 1B). Transmission electron microscopy showed clear differences in leaf ultrastructure between chlorotic and shaded green leaves (Figure 1C,D). Compared with green leaves (Figure 1C), chlorotic leaves showed chloroplasts with abnormal structural development—thylakoids were observed, but the stacks of grana were not found (Figure 1D).

**Figure 2:**
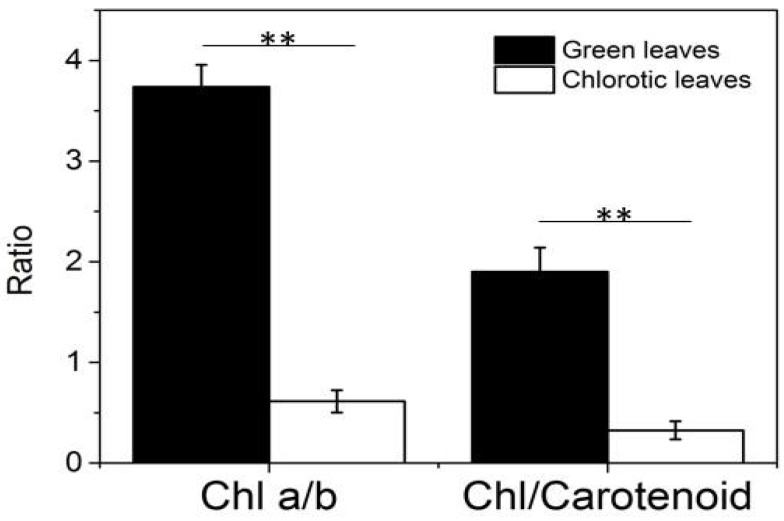
Ratio of chlorophyll a to chlorophyll b and that of total chlorophyll to carotenoids. Values represent mean ± SD of three biological replicates (** *p* < 0.01).

The contents of chlorophyll a, chlorophyll b, total chlorophyll, and carotenoids in chlorotic mutants and green leaves has been reported previously [3]. In this study, the contents of these four pigments were significantly lower in chlorotic leaves than those in green leaves. Furthermore, the ratio of chlorophyll a to chlorophyll b and that of total chlorophyll to carotenoids were significantly lower in chlorotic leaves than in green leaves (Figure 2).

Leaf gas exchange analysis showed that net photosynthesis and intercellular CO_2_ concentration were reduced by approximately 21.7% and 36.13%, respectively, in chlorotic leaves compared with green leaves. By contrast, the stomatal conductance and transpiration rate of chlorotic leaves were increased by approximately 15.2% and 21.4%, respectively, compared with green leaves (Table 1).

### 2.2. Quantitative Identification of Tea Leaf Proteins Using iTRAQ

Differentially accumulated proteins in chlorotic and green leaves were identified using iTRAQ technique, and 302,042 spectra were obtained. Analysis using the Mascot software revealed that the number of matched spectra and unique spectra were 15,804 and 14,943, respectively. A total of 6157 unique peptides were identified. Distributions of protein mass, peptide number, and peptide length are shown in Supplementary Figures S1–S3.

**Figure 3:**
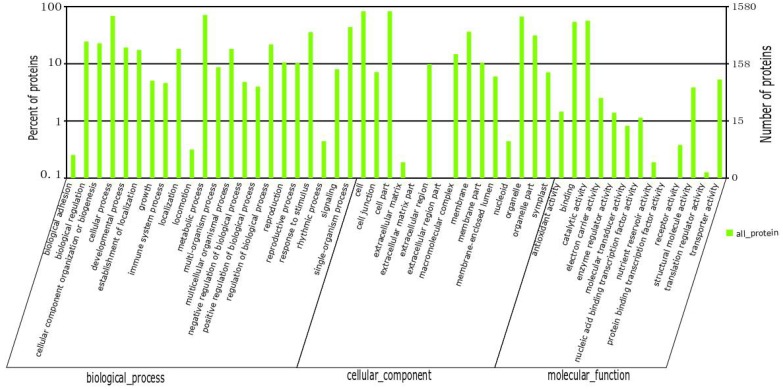
Gene ontology (GO) classification of differentially accumulated proteins in chlorotic and green leaves of the tea plant mutant cultivar “Huangjinya”.

**Figure 4:**
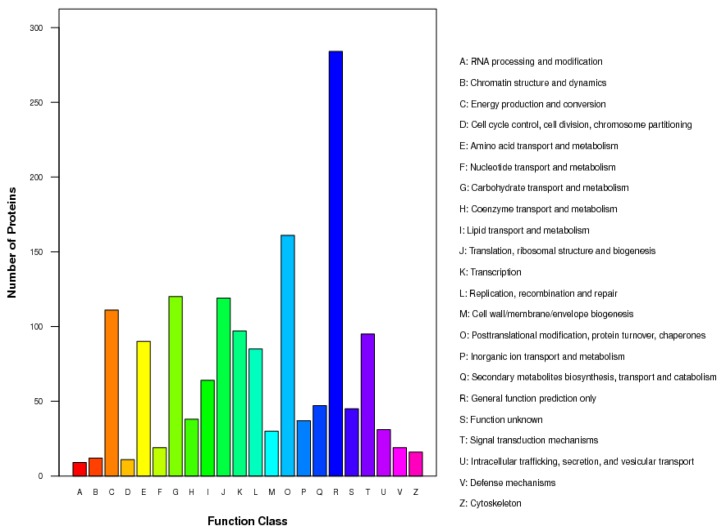
Clusters of Orthologous Groups of proteins (COG) classification of differentially accumulated proteins in chlorotic and green tea leaves of the tea plant mutant cultivar “Huangjinya”. A: RNA processing and modification; B: Chromatin structure and dynamics; C: Energy production and conversion; D: Cell cycle control, cell division, chromosome partitioning; E: Amino acid transport and metabolism; F: Nucleotide transport and metabolism; G: Carbohydrate transport and metabolism; H: Coenzyme transport and metabolism; I: Lipid transport and metabolism; J: Translation, ribosomal structure, and biogenesis; K: Transcription; L: Replication, recombination, and repair; M: Cell wall/membrane/envelope biogenesis; O: Post-translational modification, protein turnover, chaperones; P: Inorganic ion transport and metabolism; Q: Secondary metabolites biosynthesis, transport, and catabolism; R: General function prediction only; S: Function unknown; T: Signal transduction mechanisms; U: Intracellular trafficking, secretion, and vesicular transport; V: Defense mechanisms; Z: Cytoskeleton.

We identified 2110 proteins. According to GO analysis, 1354, 1284, and 1349 proteins were annotated as cellular components, functional molecules, and those involved in biological processes, respectively (Figure 3). The main biological function categories included nucleoside phosphate metabolic process, photosynthesis, and carbohydrate derivative catabolic process. The proteins classified as having functional molecular properties were mainly classified based on their activity: hydrolase activity, acting on glycosyl bonds, alpha-glucosidase activity, translation elongation factor activity, glucosidase activity, ATP-dependent peptidase activity, and oxidoreductase activity. A total of 1540 proteins were assigned to 22 categories using the Clusters of Orthologous Groups of proteins (COG) database; the main functional categories were transport and metabolism (21.5%); protein turnover, chaperones (10.5%); energy production and conversion (7.2%); and translation, ribosomal structure, and biogenesis (7.7%) (Figure 4). Additionally, 1268 proteins were annotated in 119 pathways using the Kyoto Encyclopedia of Gene and Genomes (KEGG) database. The main pathways were metabolic pathways (30.52%); biosynthesis of secondary metabolites (17.03%); plant–pathogen interaction (4.18%); protein processing in endoplasmic reticulum (3.79%); starch and sucrose metabolism (3.39%); and pyruvate metabolism (3.15%) (Supplementary Table S1).

### 2.3. Regulation of Proteins in Response to Chlorosis

In this study, 173 proteins showed significant difference (ratio of protein abundance > 1.2/0.8 fold; *p* < 0.05) between chlorotic and green leaves, including 80 up-regulated and 93 down-regulated proteins (Table 2). A total of 23, 49, and 87 proteins reproducibly decreased by 0.50-, 0.67-, and 0.83-fold, respectively, in chlorotic leaves compared with green leaves (Supplementary Table S2). On the other hand, levels of 3, 23, and 75 proteins increased by more than 2.0-, 1.5-, and 1.2-fold, respectively, in chlorotic leaves compared to in green leaves (Supplementary Table S3).

KEGG pathway enrichment analysis was employed to explore the metabolic and biosynthetic pathways, which changed in response to the chlorotic mutation with those differentially accumulated proteins. A number of such pathways were identified, including chlorophyll biosynthesis, carbohydrate transport and metabolism, energy production and conversion, flavonoid metabolism, nitrogen metabolism, chloroplast function, and oxidative stress (Table 3).

The differentially accumulated proteins involved in chlorophyll biosynthesis included nine proteases. The up-regulation of six of these proteins, including glutamyl-tRNA (Gln) amino-transferase, geranylgeranyl, glutaminyl-tRNA synthetase, magnesium chelatase, magnesium protoporphyrin, and porphobilinogen deaminase, was increased in the chlorotic leaves than in green leaves by 1.02- to 1.68-fold, whereas that of the remaining three proteins, including chlorophyll(ide) b reductase, protochlorophyllide reductase, and violaxanthin de-epoxidase, was reduced in the chlorotic leaves by 0.8- to 0.93-fold.

The differentially accumulated proteins related to carbohydrate transport and metabolism mainly comprised six rate-limiting enzymes of the glycolytic pathway (6-phosphofructokinase, fructokinase, hexokinase, pyruvate kinase, phosphoglycerate mutase, and phosphopyruvate hydratase), five glycosidases (beta-fructofuranosidase, xylosidase, galactose oxidase, UDP-L-arabinosidase, and beta-glucosidase), and three proteases related to photosynthesis (ribulose-bisphosphate carboxylase, fructose-1,6-bisphosphatase, and granule-bound starch synthase). The level of eight proteins was up-regulated by 1.01- to 1.77-fold in the chlorotic leaves. Additionally, the level of six proteins was down-regulated by 0.38- to 0.99-fold in the chlorotic leaves.

The most relevant pathway for energy generation and conversion is the tricarboxylic acid (TCA) cycle or the Krebs cycle, which generates the highest amount of energy in the most efficient way through the oxidation of sugars and other substances. The differentially accumulated proteins involved in the Krebs cycle mainly include five dehydrogenases (isopropyl-malate dehydrogenase, dihydrolipoyl dehydrogenase, malate dehydrogenase, pyruvate dehydrogenase, and succinate dehydrogenase), two dihydrolipoyllysine-residue transferases (dihydrolipoyllysine-residue acetyltransferase and dihydrolipoyllysine-residue succinyltransferase), the other are ATP citrate synthases and aconitate hydratase. Among these proteins, 16 were up-regulated by 1.05- to 1.48-fold and 4 were down-regulated by 0.95- to 0.76-fold in the chlorotic mutation compared to green leaves.

Of the eleven differentially accumulated proteins involved in flavonoid metabolism, the levels of eight proteins (cinnamate acid 4-hydroxylase (C4H), chalcone isomerase (CHI), chalcone synthase (CHS), anthocyanidin synthase (ANS), anthocyanidin reductase (ANR), anthocyanidin 3-*O*-glucosyltransferase (A3Glc), 3-dehydroshikimate dehydratase (3DSD), and 3-dehydroquinate synthase (3DHQ)) were increased by 1.09- to 1.84-fold in chlorotic leaves than in green leaves, whereas those of three proteins (phenylalanine ammonia-lyase (PAL), 4-coumarate-CoA ligase (4CL), and flavonol synthase (FLS)) were reduced by 0.62- to 0.75-fold in the chlorotic leaves compared to in green leaves. Phenylalanine is a precursor of the flavonoid biosynthesis pathway, and PAL and 4CL play key roles in the conversion of phenylalanine to coumaroyl CoA. Our results showed that levels of PAL and 4CL proteins were reduced in the chlorotic leaves, indicating that flavonoid metabolism was inhibited. From the branching of flavonoid biosynthesis (i.e., the synthetic pathway of anthocyanins and flavonols), the accumulation of anthocyanins was promoted and the synthesis of flavonols was inhibited.

Among the proteins involved in nitrogen metabolism, the levels of nine proteins’ expression (3-deoxy-7-phosphoheptulonate synthase, alanine transaminase, anthranilate synthase, aspartate kinase, cysteine synthase, glutamate synthase, homoserine kinase, methionine synthase, and glutathione reductase, GR [NADPH; nicotinamide adenine dinucleotide phosphate]) were increased and the expression levels of three proteins (ferredoxin-nitrite reductase, glycine hydroxymethyl transferase, and S-adenosyl methionine synthase) were reduced. These results indicated that the up-regulation of the expression of most amino acid synthase proteins might promote nitrogen assimilation and recycling.

Photosystem Q(B) plays an important role in chloroplast function, and the expression level of its protein was down-regulated by 0.4-fold in chlorotic leaves compared with green leaves, indicating that chloroplast function was inhibited under strong light stress. Dehydrins, also known as LEA D-11 or LEA II, are proteins whose expression is induced by various environmental stress factors [13]. B5TV66_CAMSI Putative dehydrin was annotated as an oxidative stress protein, and its expression level was significantly up-regulated by 2.29-fold. Therefore, it is speculated that the antioxidant capacity of chlorotic tea leaves might be enhanced compared to that of green leaves.

### 2.4. Integrated Analysis of Transcriptomic and Proteomic Datasets

A total of 5051 differentially expressed genes (DEGs), with differences between chlorotic and shaded green leaves, were selected for bioinformatics analysis. The combination of transcriptomic and proteomic datasets revealed correlations between 126, 52, and 19 genes and proteins in identification, quantitation, and differential expression levels, respectively (Table 4). Nineteen genes and proteins with significant differences at the quantitative level are shown in Table 5. These were classified in the following categories: chloroplast structure and function; carbohydrate and amino acid metabolism; and flavonoid biosynthesis and oxidative stress.

Genes and proteins related to chloroplast structure and function are related to the chloroplast stroma thylakoids (i.e., V-type proton ATPase subunit C, tRNA (cytosine38-C5)-methyltransferase, chloroplast small heat shock protein). The C metabolism pathway mainly involves proteins for starch synthesis (glycogen synthase), alpha-maltase (alpha-glucosidases), and lignin metabolism (l-ascorbate oxidase). Metabolic processes associated with nitrogen metabolism are mainly arginine metabolism (arginase). Oxidative stress caused by the etiolating mutation of tea leaves was also related to gene expression and protein accumulation levels. Oxidative stress-related proteins and enzymes changed significantly (fructose-bisphosphate aldolase and dehydrin). The change of the polyphenol metabolic pathway is mainly related to its upstream pathway, such as 4-coumarate-CoA ligase (CL48129Contig1), phenylalanine ammonia lyase (comp64735_c0_seq1_2), two key genes and enzyme levels were significantly down-regulated in etiolated leaves.

## 3. Discussion

Carbohydrates are a direct product of carbon assimilation via photosynthesis. Additionally, carbohydrates represent a source of plant energy and are involved in the formation of the plant cytoskeleton. In this study, the increased stomatal conductance and the decreased CO_2_ concentration was accompanied by a reduced net photosynthesis rate in the chlorotic leaves. However, the concentration of CO_2_ in the study of *Brassica napus* increased [14], possibly because of differences in the leaf structure of the two plants. Both studies have shown impaired carbon fixation efficiency in chlorotic leaves. In this study, levels of some proteins involved in carbohydrate metabolism, including fructokinase, hexokinase, phospho-pyruvate hydratase, ribulose-bisphosphate carboxylase, granule-bound starch synthase, and xylosidase, were reduced, which is consistent with this speculation. Rubisco (ribulose-bisphosphate carboxylase/oxygenase) is the rate-limiting enzyme for carbon fixation in photosynthetic reactions, and is essential for improving the photosynthetic efficiency of plants [15]. In this study, the abundance of Rubisco protein was significantly reduced in chlorotic leaves compared with green leaves (Table 3), leading to a reduction in carbohydrate biosynthesis and sugar content. Simultaneously, proteins (6-phosphate fructokinase, pyruvate kinase, phosphoglycerate mutase, and fructose 1,6-bisphosphatase) with higher expression levels were involved in the glycolytic pathway in chlorotic leaves, promoting carbohydrate catabolism. Some scholars have shown that chloroplast endometrial damage activates the expression of glycolysis-related genes [4]. Results of protein accumulation and gene expression indicated that carbohydrate accumulation was decreased in “Huangjinya” leaves under strong light.

Carbon and nitrogen metabolism balance guarantees the normal growth of tea plants, and enhanced nitrogen metabolism is accompanied by the reduced capability of photosynthesis and carbon metabolism [4]. In this study, methionine synthetase, cysteine synthetase, and glutamate synthetase were up-regulated in chlorotic leaves compared with green leaves, indicating that strong light promotes nitrogen metabolism and amino acid accumulation in “Huangjinya”, which is consistent with a previous study [16]. We speculate that reduced chlorophyll biosynthesis in turn reduced nitrogen consumption, thus increasing the content of upstream substances (amino acids) in leaves. In addition, environmental stress such as intense light and high temperature affect the normal growth of plants, and reactive oxygen species (ROS) are accumulated as a by-product [17]. Previous studies have shown that increasing the glutathione reductase activity in chloroplasts improves the photochemical ratio in transgenic cotton plants, thereby reducing photoinhibition [18]. This suggests that enhanced glutathione reductase activity protects the leaf cell biofilm and enhances the plant’s ability to defend itself against abiotic stress (e.g., UV-B radiation) [19]. In this study, the expression of glutathione reductase protein was increased by 1.3-fold, and as a result the ability to remove ROS produced by UV-B radiation under intense light might be enhanced.

**Figure 5:**
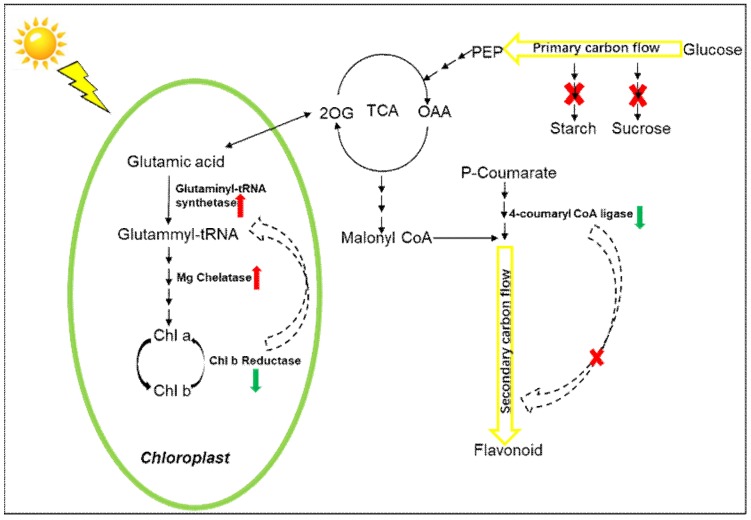
A simplified schematic presentation of the weakening of carbon metabolism in the chlorotic mutation. Red arrows indicate up-regulated expression of the protein. Green arrows indicate down-regulated expression of the protein. Black solid arrows indicate metabolic pathways. Black dotted arrows indicate regulatory relationships between metabolites and metabolites or metabolites and metabolic pathways. Red cross indicates inhibition of related metabolic pathways.

A simplified schematic presentation of the weakening of carbon metabolism in the chlorotic mutation is shown in Figure 5. The ratio of chlorophyll a to chlorophyll b has been considered as an important parameter to measure the light tolerance of plants. An increase in this ratio is beneficial for the absorption of blue-violet light, which is suitable for plant growth in the dark [20]. Generally, the biosynthesis and degradation of chlorophyll a and chlorophyll b in plants occur in a dynamic cycle, and chlorophyll biosynthesis requires relatively low light intensity. However, as a light-sensitive plant, the ratio of chlorophyll a to chlorophyll b in leaves of “Huangjinya” under the shade was significantly higher than that under intense light, suggesting that the conversion of chlorophyll a to chlorophyll b is accelerated under strong light. Increase in chlorophyll b content is beneficial for the plant’s adaptation to light stress. Chlorophyll(ide) b reductase is the key enzyme that catalyzes the first step in chlorophyll b degradation, and plays an important role in the process of leaf senescence, with the degradation of LHC-II and chloroplast matrix [21]. Studies have reported that chlorophyll b reductase was involved in the conversion process of chlorophyll b and chlorophyll a, which is considered to be an important clue for chlorophyll b degradation [22]. In this study, the level of chlorophyll(ide) b reductase was reduced by 0.9-fold in chlorotic leaves, and the ratio of chlorophyll a to chlorophyll b was lower in chlorotic leaves than in green leaves, indicating that the activity of chlorophyll b reductase was suppressed. Zhang and Tan [23] also obtained similar results regarding the reduction of chlorophyll content and chlorophyll a/b ratio under salt stress. The studies of Sang et al. [24] and Huang et al. [25] suggested that the decreased ratio of chlorophyll a/b resulted from a higher sensitivity to various environmental stresses for chlorophyll a compared to chlorophyll b. Therefore, the pathway of chlorophyll b biosynthesis is still enhanced although the total chlorophyll content was decreased in the chlorotic leaves. Interestingly, the content of chlorophyll b was not increased with the enhanced pathway of chlorophyll b synthase in “Huangjinya” leaves under strong light. Because the process of chlorophyll biosynthesis is complex and highly conserved, the mutation of a gene can severely affect the chlorophyll content, leading to a different color phenotype of leaves [26]. Magnesium chelatase is another enzyme with a significant effect on chlorophyll biosynthesis. It catalyzes the insertion of Mg^2+^ into protoporphyrin IX [27]. In the process of leaf albinism, the abundance of magnesium chelatase subunit proteins in higher plants is significantly increased under light [28,29]. In this study, the expression level of magnesium chelatase protein was higher in chlorotic leaves by 1.34-fold than in green leaves, which is consistent with the study of Walker et al. [29]. Müller et al. [30] showed that oxidative stress improved the quality of monomeric chlorophyll H in *Escherichia coli*. Moreover, genes involved in the biosynthesis of chlorophyll were induced by ROS in “Huangjinya” leaves, but the sub-structure of magnesium chelatase was not influenced, although its activity was enhanced. Comprehensively, we proposed that a feedback mechanism existed in weakened carbon metabolism, where the pathway of chlorophyll b biosynthesis was positively regulated as a result of increased expression of Mg chelatase proteins and decreased expression of chlorophyll b reductase proteins.

Flavonoids are important secondary metabolites of carbon metabolism in the tea plant, and have several physiological functions. Flavonoids provide plants with vibrant pigmentation, which protects the plants from UV-B radiation and helps attract pollinators as well as seed dispersers [31]. Because photosynthetic carbon assimilation is severely inhibited in chlorotic leaves, flavonoid biosynthesis is accordingly reduced [32]. Similar to the results of Zhang et al. [2], in this study, the expression levels of PAL and 4CL proteins (i.e., the rate-limiting enzymes of the flavonoid biosynthesis pathway) were down-regulated by 0.70- and 0.75-fold in chlorotic leaves (Table 3). Additionally, changes in the gene expression of 4CL confirmed the result of 4CL protein expression, thereby inhibiting the accumulation of flavonoids. A previous study showed that most of flavonoids were accumulated on the leaf surface to protect structure and organization in plants from UV-B radiation damage [33], while in this study, our results indicated that abnormally developed chloroplast inhibited the accumulation of chlorophyll and flavonoids because few carbon skeletons were provided as a result of weakening of carbon metabolism, and the reduction of flavonoids contents was unfavorable for the chlorotic mutant to protect against UV-B radiation damage.

## 4. Materials and Methods

### 4.1. Plant Material and Shading Treatment

“Huangjinya” (*Camellia sinensis* (L.) *O. Kuntze* cv.) is a light-sensitive albino tea variety, which displays yellow shoots under strong light condition. The low levels of flavonoids in “Huangjinya” reveals that the metabolism of flavonoids has great correlation to strong light stress [1,2]. A mutant of “Huangjinya” was officially released in Zhejiang province in 2008 and specimens were obtained free of charge from the owner of the mutant Deshi Tea Plantation, Yuyao, Zhejiang province [34]. Then, the experimental material was planted in pots at the Tea Research Institute, Chinese Academy of Agricultural Sciences (TRI, CAAS), Hangzhou, China. In March 2014, 60 pots of tea plants with uniform young shoots, with one bud and one leaf, were selected for the experiment. Half of the pots were treated with high-density polyethylene tape with two-pin net (60% sun-shading, 320–800 µmol·m^−2^·s^−1^), and the remaining half were exposed to full sunlight (800–2000 µmol·m^−2^·s^−1^) for ten days. Randomly selected samples of young shoots, with one bud and two leaves, were harvested, immediately frozen in liquid nitrogen, and stored in a −70 °C ultra-refrigerator. Sampling was repeated six times from shaded and unshaded plants.

### 4.2. Electron Microscope Analysis

Transmission electron microscope (TEM, Hitachi Ltd., Tokyo, Japan) was used to observe the ultrastructure of chlorotic leaves. Leaf samples (approximately 1 mm^2^) were fixed in 2.5% glutaraldehyde solution overnight at 4 °C. Ultrathin sections of fixed leaves were cut, stained, and viewed under a JEM-1230 transmission electron microscope (Nippon Tekno, Tokyo, Japan) at an accelerating voltage of 80 kV as described previously [1].

### 4.3. Leaf Gas Exchange Measurement

The fifth leaf of potted chlorotic mutants and wild-type tea plants was subjected to gas exchange analysis. A Li-Cor 6400 portable photosynthesis system (Li-Cor Inc., Lincoln, NE, USA) with a built-in light source set at 1000 μmol photons·m^−2^·s^−1^ was used to determine the net photosynthesis and stomatal conductance. All measurements were carried out between 09:00 a.m. and 11:00 a.m., with the leaf temperature adjusted to 25 °C.

### 4.4. Protein Extraction, iTRAQ Labeling, Data Acquisition, and Processing

Samples used for proteomic analysis were the same as those used for transcriptome analysis, and consisted of two biological replicates of shaded and unshaded leaves. All of the steps, including protein extraction, iTRAQ labeling of protein samples, liquid chromatography electrospray ionization tandem mass spectrometry (LC-ESI-MS/MS) analysis based on Q Exactive, mass spectrometer data analysis, and functional annotation of proteins, were performed as described previously [35]. Protein identification was performed using the Mascot search engine (Matrix Science, London, UK; version 2.3.02) against a database containing 133,175 sequences.

### 4.5. Quantitative Real-Time PCR (qRT-PCR) Analysis

Total RNA was isolated using an RNA plant plus kit (Tiangen, China). cDNA was synthesized using a Prime Script TM RT reagent Kit (TaKaRa, Biotechnology Co., Ltd., Dalian, China). qRT-PCR was performed on the Applied Biosystems 7300 machine (Carlsbad, CA, USA). Primer pairs used for qRT-PCR are shown in Supplementary Table S4, and GAPDH was used as the reference gene. For each target gene, triplicate reactions were performed. Relative transcript levels were calculated against that of the internal control (GAPDH) according to the equation 2^−ΔΔ*C*t^. All data are shown as mean ± standard deviation (SD) (*n* = 3).

### 4.6. Bioinformatics Analysis

Functional analysis of the identified proteins was conducted using gene ontology (GO) annotation (Available online: http://www.geneontology.org/), and proteins were categorized according to their biological process, cellular components, and molecular function. The differentially accumulated proteins were further classified into the Clusters of Orthologous Groups of proteins database (Available online: http://www.ncbi.nlm.nih.gov/COG/) and Kyoto Encyclopedia of Gene and Genomes (KEGG) database (Available online: https://www.kegg.jp/kegg/pathway.html). GO and pathway enrichment analyses were performed to determine the functional sub-categories and metabolic pathways in which the differentially accumulated proteins showed significant enrichment. Cluster analysis of differentially accumulated proteins was performed using Cluster 3.0 (Stanford University, California, USA).

Data of integrated transcriptomic analysis reported previously [3] were deposited in the Sequence Read Archive (SRA) database (Available online: https://trace.ncbi.nlm.nih.gov/Traces/sra/) of the National Center for Biotechnology Information (NCBI) under the accession number SRP072792.

## 5. Conclusions

In this study, 2110 proteins were identified in “Huangjinya” leaves, the expression levels of 19 of which changed significantly, correlating with RNA expression. Differential protein expression analysis indicated that primary carbon metabolism (i.e., carbohydrate synthesis and transport) was inhibited in chlorotic tea leaves. The differentially expressed genes and proteins combined with photosynthetic phenotypic data suggested that 4-coumarate-CoA ligase (4CL) had a major effect on repressing flavonoid metabolism (secondary carbon metabolism), and abnormal developmental chloroplast inhibited the accumulation of chlorophyll and flavonoids because few carbon skeletons were provided as a result of the weakened primary carbon metabolism. Additionally, a positive feedback mechanism was verified at the protein level (Mg chelatase and chlorophyll b reductase) in the chlorophyll biosynthetic pathway, which might effectively promote the accumulation of chlorophyll b in response to the demand for this pigment in the cells of chlorotic tea leaves in weakened carbon metabolism.

## Figures and Tables

**Table 1:** Leaf gas exchange analysis of green and chlorotic leaves of the tea plant mutant cultivar “Huangjinya”.

Genotype	NPR ^a^ (μmol CO_2_·m^−2^·s^−1^)	SC ^b^ (mmol H_2_O·m^−2^·s^−1^)	IC ^c^ (μmol CO_2_·mol^−1^)	TR ^d^ (mmol H_2_O·m^−2^·s^−1^)
Green	8.17 ± 0.45	0.046 ± 8.39 × 10^−4^	256.54 ± 18.04	1.45 ± 0.07
Chlorotic	6.39 ± 0.25 **	0.053 ± 5.1 × 10^−3^ *	163.85 ± 14.28 **	1.76 ± 0.13 **

^a^ net photosynthetic rate; ^b^ stomatal conductance; ^c^ intercellular CO_2_ concentration; ^d^ transpiration rate (**: *p* < 0.01; *: *p* < 0.05).

**Table 2:** Pathway enrichment analysis of differentially accumulated proteins in green and chlorotic leaves of the tea plant mutant cultivar “Huangjinya”.

Pathway	Pathway	Enrichment Score	Number of Proteins	
ID ^a^		Scores ^b^	Up ^c^	Down ^d^
ko00195	Photosynthesis	0.003016	0	6
ko00603	Glycosphingolipid biosynthesis—globo series	0.010268	1	2
ko00052	Galactose metabolism	0.016302	2	3
ko00600	Sphingolipid metabolism	0.030562	2	1
ko00511	Other glycan degradation	0.031593	2	2
ko00190	Oxidative phosphorylation	0.034353	2	5
ko00604	Glycosphingolipid biosynthesis—ganglio series	0.059274	1	1
ko00531	Glycosaminoglycan degradation	0.059274	1	1
ko00904	Diterpenoid biosynthesis	0.107256	0	1
ko03040	Spliceosome	0.133227	2	6
ko00906	Carotenoid biosynthesis	0.167604	1	1
ko00402	Benzoxazinoid biosynthesis	0.203083	0	1
ko01110	Biosynthesis of secondary metabolites	0.208389	17	10
ko00330	Arginine and proline metabolism	0.241617	1	2
ko04144	Endocytosis	0.251564	1	3
ko04145	Phagosome	0.276463	0	4
ko03010	Ribosome	0.284144	5	2
ko00062	Fatty acid elongation	0.288692	1	0
ko00710	Carbon fixation in photosynthetic organisms	0.301754	4	0
ko00520	Amino sugar and nucleotide sugar metabolism	0.327304	0	4
ko00900	Terpenoid backbone biosynthesis	0.333655	2	0
ko00770	Pantothenate and CoA biosynthesis	0.365164	1	0
ko00196	Photosynthesis—antenna proteins	0.365164	0	1
ko00730	Thiamine metabolism	0.365164	0	1
ko00300	Lysine biosynthesis	0.365164	1	0
ko04141	Protein processing in endoplasmic reticulum	0.411499	4	2
ko00460	Cyanoamino acid metabolism	0.414401	1	1
ko01100	Metabolic pathways	0.418741	21	22
ko00051	Fructose and mannose metabolism	0.425589	2	1
ko03030	DNA replication	0.433469	1	0
ko00073	Cutin, suberine, and wax biosynthesis	0.433469	0	1
ko00480	Glutathione metabolism	0.452965	2	0
ko00561	Glycerolipid metabolism	0.490057	1	1
ko00450	Selenocompound metabolism	0.494474	1	0
ko00500	Starch and sucrose metabolism	0.497233	3	2
ko01040	Biosynthesis of unsaturated fatty acids	0.548952	1	0
ko00130	Ubiquinone and other terpenoid-quinone biosynthesis	0.548952	0	1
ko00940	Phenylpropanoid biosynthesis	0.551185	1	3
ko00360	Phenylalanine metabolism	0.591425	0	2
ko00860	Porphyrin and chlorophyll metabolism	0.597598	1	0
ko00562	Inositol phosphate metabolism	0.597598	1	0
ko00350	Tyrosine metabolism	0.597598	1	0
ko00020	Citrate cycle (TCA cycle)	0.621742	2	0
ko00240	Pyrimidine metabolism	0.641032	1	0
ko00592	alpha-Linolenic acid metabolism	0.679808	1	0
ko00280	Valine, leucine, and isoleucine degradation	0.679808	1	0
ko00290	Valine, leucine, and isoleucine biosynthesis	0.679808	1	0
ko00400	Phenylalanine, tyrosine, and tryptophan biosynthesis	0.714424	1	0
ko03015	mRNA surveillance pathway	0.725342	2	1
ko00941	Flavonoid biosynthesis	0.745321	0	1
ko00910	Nitrogen metabolism	0.745321	0	1
ko03013	RNA transport	0.751412	5	0
ko00250	Alanine, aspartate, and glutamate metabolism	0.772898	1	0
ko00061	Fatty acid biosynthesis	0.797508	1	0
ko00640	Propanoate metabolism	0.797508	1	0
ko00010	Glycolysis/gluconeogenesis	0.808197	3	0
ko00071	Fatty acid metabolism	0.819469	1	0
ko03018	RNA degradation	0.821126	2	0
ko00620	Pyruvate metabolism	0.822001	3	0
ko00230	Purine metabolism	0.856546	1	0
ko00030	Pentose phosphate pathway	0.856546	1	0
ko03008	Ribosome biogenesis in eukaryotes	0.886052	1	0
ko04146	Peroxisome	0.909525	1	0
ko00053	Ascorbate and aldarate metabolism	0.909525	0	1
ko04075	Plant hormone signal transduction	0.951118	0	2
ko04626	Plant–pathogen interaction	0.997861	1	0

^a^ Serial number of the enrichment pathway of differential accumulated proteins in the Kyoto Encyclopedia of Gene and Genomes (KEGG); ^b^ Degree of pathway enrichment of differential proteins in KEGG; ^c^ Number of up-accumulated proteins; ^d^ Number of down-accumulated proteins.

**Table 3:** Differentially accumulated proteins identified in pathways potentially associated with chlorophyll deficiency in green and chlorotic leaves of the tea plant mutant cultivar “Huangjinya”.

Identity Proteins ^a^	EC Number ^b^	Accession ^c^	Fold Change (Etiolation/Green)
**Chlorophyll biosynthesis**			
Aspartyl-tRNA/glutamyl-tRNA amidotransferase subunit A	6.3.5.6	CL57658Contig1	1.12
Chlorophyll(ide) b reductase	1.1.1.294	CL49902Contig1	0.90
Geranylgeranyl	2.5.1.1	CL1Contig45	1.41
Glutaminyl-tRNA synthetase	6.1.1.18	CL18599Contig1	1.26
Magnesium chelatase	6.6.1.1	CL498Contig6	1.34
Magnesium protoporphyrin	2.1.1.11	CL18563Contig1	1.02
Porphobilinogen deaminase	2.5.1.61	CL37040Contig1	1.68
Protochlorophyllide reductase	1.3.1.33	CL508Contig2	0.80
Violaxanthin de-epoxidase	1.10.99.3	CL3Contig71	0.93
**Carbohydrate transport and metabolism**			
6-Phosphofructokinase	2.7.1.11	CL128Contig12	1.01
Fructokinase	2.7.1.4	CL18457Contig1	0.99
Hexokinase	2.7.1.1	CL60051Contig1	0.88
Phosphoglycerate mutase	5.4.2.1	CL15710Contig1	1.24
Phosphopyruvate hydratase	4.2.1.11	CL19736Contig1	0.95
Pyruvate kinase	2.7.1.40	comp42454_c0_seq1_3	1.56
Ribulose-bisphosphate carboxylase	4.1.1.39	CL8Contig62	0.76
Granule-bound starch synthase		CL7825Contig1	0.38
Fructose-1,6-bisphosphatase		comp51045_c1_seq8_2	1.72
Beta-fructofuranosidase		CL53580Contig1	1.26
Xylosidase		comp80972_c0_seq1_4	0.99
Galactose oxidase		CL17517Contig1	1.23
UDP-l-arabinosidase		comp62280_c0_seq4_2	1.19
Beta-glucosidase		CL167Contig8	1.77
**Energy production and conversion**			
Aconitate hydratase	4.2.1.3	CL79359Contig1	1.37
ATP-citrate synthase	2.3.3.1	comp116390_c0_seq1_3	0.95
Dihydrolipoyl dehydrogenase	1.8.1.4	comp99158_c0_seq16_4	0.86
Dihydrolipoyllysine-residue acetyltransferase	2.3.1.12	CL17321Contig1	1.47
Dihydrolipoyllysine-residue succinyltransferase	2.3.1.61	CL64635Contig1	0.78
Isopropylmalate dehydrogenase	1.1.1.85	comp102244_c2_seq1_4	1.11
Malate dehydrogenase		CL2510Contig4	1.22
Pyruvate dehydrogenase	1.2.4.1	CL37234Contig1	1.31
Succinate dehydrogenase	1.3.5.1	comp131171_c0_seq3_3	1.48
**Flavonoid metabolism**			
4-Coumarate-CoA ligase	6.2.1.12	CL48129Contig1	0.75
Anthocyanidin 3-*O*-glucosyltransferase	2.4.1.115	CL319Contig5	1.13
Anthocyanidin reductase	1.3.1.77	CL103Contig2	1.09
Anthocyanidin synthase	1.14.11.19	CL3972Contig1	1.48
Chalcone isomerase	5.5.1.6	CL12172Contig2	1.49
Chalcone synthase	2.3.1.74	CL8845Contig1	1.84
Cinnamate 4-hydroxylase	1.14.13.11	CL30220Contig1	1.05
Flavonol synthase	1.14.11.23	CL11177Contig1	0.62
Phenylalanine ammonia-lyase	4.3.1.24	comp64735_c0_seq1_2	0.70
3-Dehydroshikimate dehydratase	4.2.1.118	CL16483Contig1	1.14
3-Dehydroquinate synthase	4.2.3.4	CL55Contig2	1.30
**Nitrogen metabolism**			
3-Deoxy-7-phosphoheptulonate synthase activity	2.5.1.54	comp124631_c0_seq3_3	1.02
Alanine transaminase	2.6.1.2	CL34278Contig1	1.52
Anthranilate synthase	4.1.3.27	CL19422Contig1	1.34
Aspartate kinase	2.7.2.4	CL43459Contig1	1.13
Cysteine synthase	2.5.1.47	CL37334Contig1	1.14
Ferredoxin-nitrite reductase	1.7.7.1	CL61698Contig1	0.69
Glutamate synthase	1.4.7.1	comp109180_c0_seq1_1	1.36
Glycine hydroxymethyltransferase	2.1.2.1	CL5210Contig1	0.98
Homoserine kinase	2.7.1.39	CL16611Contig1	1.18
Methionine synthase	2.1.1.13	CL9637Contig1	1.19
S-adenosylmethionine synthase	2.5.1.6	CL39736Contig1	0.93
Glutathione reductase (NADPH)	1.8.1.7	CL9366Contig1	1.30
**Chloroplast function**			
Proton ATPase subunit C		CL6Contig55	0.78
Elongation factor G, chloroplastic		CL107Contig12	1.26
Protein ABCI7, chloroplastic		comp100064_c2_seq1_1	1.45
Pentatricopeptide repeat-containing protein At4g16390, chloroplastic		CL9Contig51	1.93
Chloroplast small heat shock protein		CL2Contig56	1.50
Photosystem Q(B) protein		comp95426_c0_seq3_4	0.40
Cytochrome P450 86A2		CL102Contig8	1.13
**Oxidative stress**			
Fructose-bisphosphate aldolase 3, chloroplastic		CL1744Contig2	1.24
Histone deacetylase HDT1		CL85545Contig1	1.20
B5TV66_CAMSI Putative dehydrin		CL14231Contig1	2.29
Peroxidase 50		CL920Contig3	1.01

^a^ Proteins identified by isobaric tag for relative and absolute quantification (iTRAQ); ^b^ Enzyme commission numbers in PDB; ^c^ Accession number of the identified proteins in the National Center for Biotechnology Information non-redundant protein sequences (NCBI-nr) database.

**Table 4:** The number of proteins and genes identified, quantified, and differentially expressed in green and chlorotic leaves of the tea plant mutant cultivar “Huangjinya”.

Group Names	Type	Number of Proteins	Number of Genes	Number of Correlations
EM ^a^ vs. NG ^b^	Identification	2110	5051	126
EM vs. NG	Quantitation	976	5051	52
EM vs. NG	Differential Expression	173	5051	19

^a^ etiolated mutation; ^b^ normal green.

**Table 5:** Genes and proteins showing significant changes between green and chlorotic leaves as determined via the integrated analysis of transcriptomic and proteomic datasets.

Accession ^a^	log2 (EM/NG)		FDR ^b^	Description	Function
	Gene	Protein			
CL14231Contig1	2.76	1.20	0.0000	Dehydrin	Oxidative stress
CL1744Contig2	−2.7	0.31	0.0010	Fructose-bisphosphate aldolase	Carbohydrate transport and metabolism
CL2031Contig2	3.02	−1.43	0.0000	l-Ascorbate oxidase	Secondary metabolites biosynthesis, transport, and catabolism
CL2Contig56	4.57	0.58	0.0000	Chloroplast small heat shock protein	Posttranslational modification, protein turnover, chaperones
CL374Contig2	−2.29	−0.62	0.0020	Zeta-carotene desaturase	Response to hormone stimulus
CL48129Contig1	−1.56	−0.42	0.0320	4-coumarate-CoA ligase 2	Lipid transport and metabolism
CL4Contig6	3.06	−0.30	0.0000	SnRK2 calcium sensor	Calcium ion binding
CL50804Contig1	−3.79	−0.69	0.0000	Cysteine protease	Posttranslational modification, protein turnover, chaperones
CL6Contig55	−4.51	−0.36	0.0000	V-type proton ATPase subunit C	Energy production and conversion
CL73512Contig1	2.25	0.45	0.0320	Alpha-glucosidases	Carbohydrate transport and metabolism
CL7825Contig1	−2.04	−1.40	0.0370	Glycogen synthase	Carbohydrate transport and metabolism
CL8494Contig2	2.26	1.58	0.0030	Unknown	Embryo development ending in seed dormancy
CL9Contig51	1.85	0.95	0.0420	tRNA (cytosine38-C5)-methyltransferase	Chloroplast organization
Comp101085_c0_seq1_1	2.02	0.39	0.0210	Alpha-glucosidases	Carbohydrate transport and metabolism
Comp55188_c0_seq1_2	−2.99	−0.84	0.0420	Serine proteases	Posttranslational modification, protein turnover, chaperones
Comp64728_c0_seq2_2	1.86	0.75	0.0140	DEAD-box ATP-dependent RNA helicase 31	Response to water deprivation
Comp64735_c0_seq1_2	−2.25	−0.51	0.0120	Phenylalanine ammonia-lyase	Amino acid transport and metabolism
Comp74393_c0_seq1_4	1.65	0.50	0.0240	Dihydroxy-acid dehydratase	Amino acid transport and metabolism
Comp96472_c0_seq1_4	−1.8	−0.69	0.0170	Arginase	Amino acid transport and metabolism

^a^ Accession number of the identified proteins in National Center for Biotechnology Information non-redundant protein sequences (NCBI-nr). database; ^b^ FDR: false discovery rate.

## Supplementary Materials

Supplementary materials can be found at http://www.mdpi.com/1422-0067/19/12/3943/s1.
