# Supplementary figures and images for: iTRAQ-Based Quantitative Proteomics Analysis Reveals the Mechanism Underlying the Weakening of Carbon Metabolism in Chlorotic Tea Leaves
Source: Int J Mol Sci. 2018 Dec 7;19(12):3943. doi: 10.3390/ijms19123943 (PMC6321456; doi:10.3390/ijms19123943)

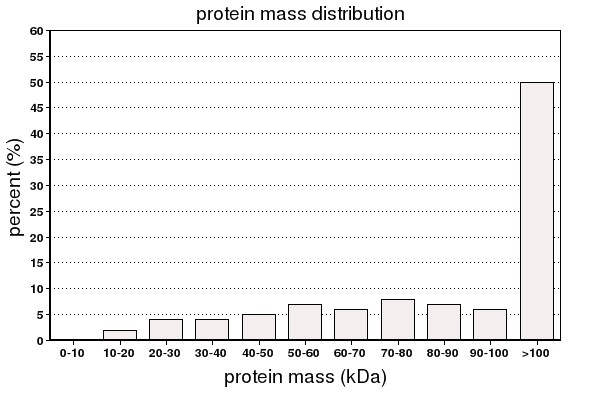

Supplement: Supplementary file 1 [file ijms-19-03943-s001.zip › Supplementary file/Supplementary Figure S1.jpg]

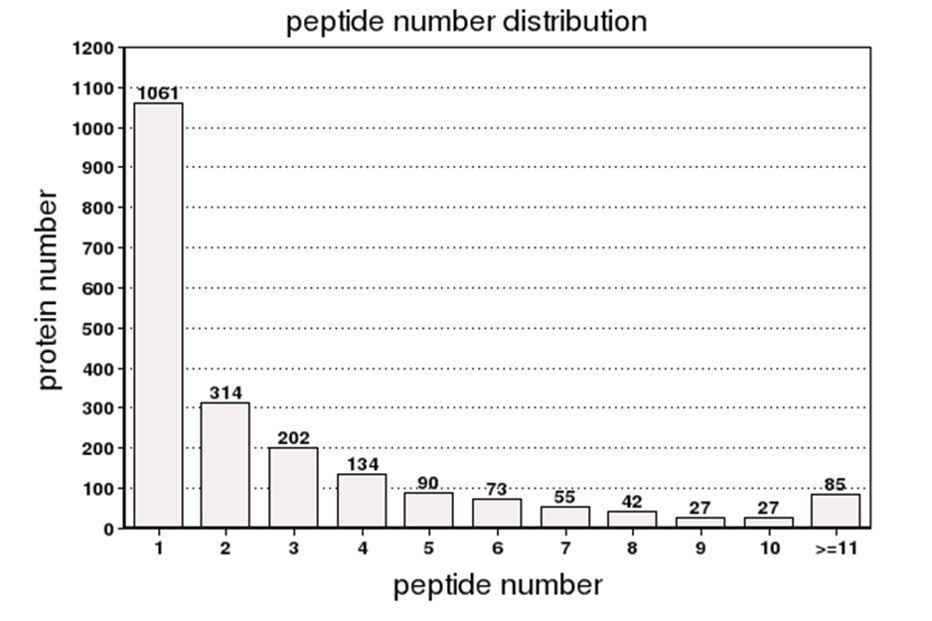

Supplement: Supplementary file 1 [file ijms-19-03943-s001.zip › Supplementary file/Supplementary Figure S2.jpg]

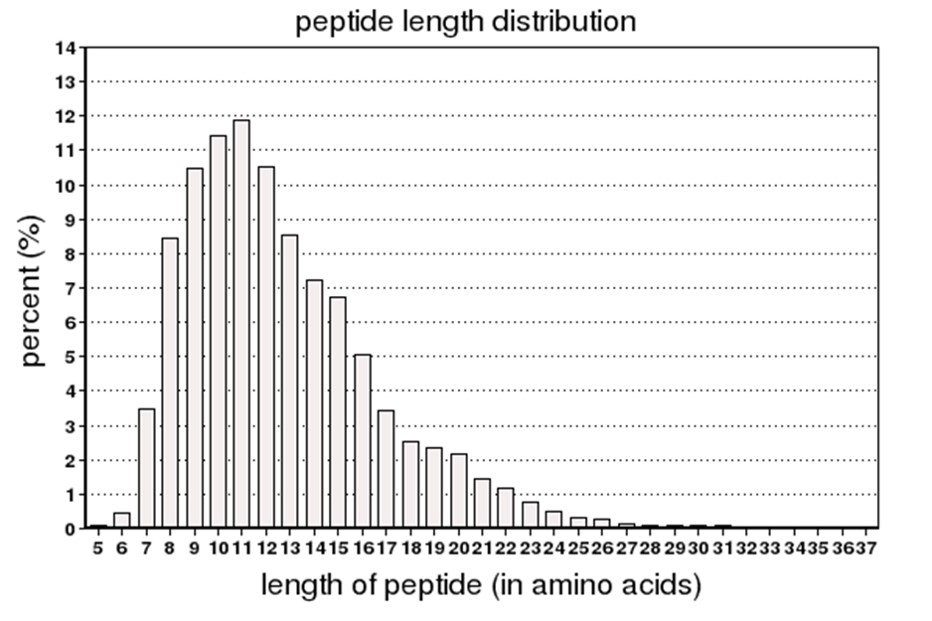

Supplement: Supplementary file 1 [file ijms-19-03943-s001.zip › Supplementary file/Supplementary Figure S3.jpg]
